# Supplementary material for: Reactive organic carbon emissions from volatile chemical products
Source: Atmos Chem Phys. Author manuscript; Available in PMC 2022 Mar 31. (PMC8193795; doi:10.5194/acp-21-5079-2021)
Supplement: Supplement1 [file NIHMS1690840-supplement-Supplement1.pdf]

Supplement of Atmos. Chem. Phys., 21, 5079–5100, 2021
https://doi.org/10.5194/acp-21-5079-2021-supplement
© Author(s) 2021. CC BY 4.0 License.

Atmospheric Chemistry and Physics
Open Access

Supplement of

Reactive organic carbon emissions from volatile chemical products

Karl M. Seltzer et al.

Correspondence to: Havala Pye (pye.havala@epa.gov)

The copyright of individual parts of the supplement might differ from the article licence.

Table S1: PUCs, sub-PUCs, NAICS codes, and SCTG codes for all sub-PUCs.

Product Use Categories (PUCs)	Sub-Product Use Categories (sub-PUCs)	NAICS Product Codes[a]	SCTG Code[b]	Producer Price Index Category[c]
Cleaning Products	Detergents & Soaps	3256111, 3256114, 3256117, 325611W	233	Soap and Other Detergent Manufacturing
	General Cleaners	3256125, 2356127, 2356121, 235611A, 2356130, 325612W	233	Polish and Other Sanitation Good Manufacturing; Soap and Other Detergent Manufacturing; Surface Active Agent Manufacturing;
Personal Care Products	Daily Use Products	3256204, 325620D, 325620G, 325620W, 3256207 (25%)[d]	232	Toilet Preparation Manufacturing
	Short Use Products	3256201, 325620A, 325611D, 3256207 (75%)[d]	232	Toilet Preparation Manufacturing; Soap and Other Detergent Manufacturing
Adhesives & Sealants	Adhesives & Sealants	3255201, 3255204, 3255207, 305520A, 325520W	239	Adhesive Manufacturing
Paints & Coatings	Architectural Coatings	3255101, 325510W	[f]	Paint and Coating Manufacturing
	Aerosol Coatings	3255107 (10%)[e]	[f]	Paint and Coating Manufacturing
	Allied Paint Products	325510B	[f]	Paint and Coating Manufacturing
	Industrial Coatings	3255104, 3255107 (90%)[e]	[f]	Paint and Coating Manufacturing
Printing Inks	Printing Inks	3259101, 3259104, 3259107, 325910A, 325910E, 325910H, 325910W	231	Printing Ink Manufacturing
Pesticides & FIFRA Products	FIFRA Pesticides	3253204, 3253207	235	Pesticide and Other Agricultural Chemical Manufacturing
	Agricultural Pesticides	3251994, 3253201, 325320W	235	All Other Basic Organic Chemical Manufacturing; Pesticide and Other Agricultural Chemical Manufacturing
Dry Cleaning	Dry Cleaning	[g]		
Oil & Gas	Oil & Gas			
Misc. Products	Misc. Products	[h]		
Fuels & Lighter	Fuels & Lighter			

[a]: NAICS Product Codes used for mapping U.S. Census Bureau (2016) ASM statistics to individual sub-PUCs.
[b]: SCTG Codes used for mapping U.S. Department of Transportation (2015) calculated commodity values to individual sub-PUCs. All values ($) and mass (tons) quantities retrieved from Table 6 of that report.
[c]: Category used when retrieving Produce Price Indices from the U.S. Bureau of Labor Statistics, FRED, Federal Reserve Bank of St. Louis.
[d]: NAICS code 3256207 includes all hair preparation products. Based on sales data by California Air Resources Board 2015 Consumer and Commercial Products Survey Data, we estimate ~75% of all hair preparation products are short-use (e.g. shampoos, conditioners) and ~25% are daily-use (e.g. hair spray, other leave-in products).
[e]: NAICS code 3255107 includes all special-purpose coating materials (e.g. automotive finishing, traffic markings, aerosolized painting products). Based on shipment data from U.S. Census Bureau, Paint and Allied Products - 2010, MA325F(10), Issued July 2011, we estimate ~10% of all special-purpose coating materials in this NAICS are aerosolized painting products, with the residual consisting of special-purpose coating materials used exclusively in industrial settings.
[f]: All Paints & Coatings commodity values retrieved from: U.S. Census Bureau, Paint and Allied Products - 2010, MA325F(10), Issued July 2011.
[g]: sub-PUC usage estimated from solvent usage as reported by The Freedonia Group; Industry Study #3429; Solvents; July 2016.
[h]: sub-PUC usage estimated from reported sales data by California Air Resources Board 2015 Consumer and Commercial Products Survey Data: https://ww3.arb.ca.gov/consprod/survey/2015_cp_survey_summary_data_2019-12-09.xlsx; last access: August 28, 2020

Table S2: PUCs, sub-PUCs, NAICS codes, shipment values, commodity price, producer price index, and product usage for all sub-PUCs (2016). Per-capita product usage calculated using the U.S. Census Bureau's 2016 estimate of the U.S. population (~323,000,000).

Product Use Categories (PUCs)	Sub-Product Use Categories (sub-PUCs)	NAICS Product Codes	ASM Shipment Values [$1000][a]	Commodity Price [$/kg][b]	2016 Producer Price Index[c]	2012 Producer Price Index[c]	Annual Usage [kg/person/year][d]	
Cleaning Products	Detergents & Soaps	3256111	7849011	1.615	1.08	1.07	14.92	40.58
		3256114	10137793		1.08	1.07	19.27	
		3256117	2740950		1.08	1.07	5.21	
		325611W	625339		1.08	1.07	1.19	
	General Cleaners	3256125	5391225	1.615	1.11	1.05	9.85	28.47
		2356127	1024203		1.11	1.05	1.87	
		2356121	1067746		1.11	1.05	1.95	
		235611A	290791		1.08	1.07	0.55	
		2356130	7173431		1.18	1.16	13.48	
		325612W	417549		1.11	1.05	0.76	
Personal Care Products	Daily Use Products	3256204	3140488	9.290	1.07	1.03	1.01	8.83
		325620D	7912985		1.07	1.03	2.54	
		325620G	13192677		1.07	1.03	4.23	
		325620W	1562269		1.07	1.03	0.50	
		3256207 (25%)[e]	1734011		1.07	1.03	0.56	
	Short Use Products	3256201	281282	9.290	1.07	1.03	0.09	3.16
		325620A	3574170		1.07	1.03	1.15	
		325611D	789937		1.08	1.07	0.26	
		3256207 (75%)[e]	5202032		1.07	1.03	1.67	
Adhesives & Sealants	Adhesives & Sealants	3255201	1405299	2.602	1.11	1.10	1.65	15.23
		3255204	7967659		1.11	1.10	9.34	
		3255207	694706		1.11	1.10	0.81	
		305520A	2405554		1.11	1.10	2.82	
		325520W	513470		1.11	1.10	0.60	
Paints & Coatings	Architectural Coatings	3255101,	11253306	2.694[g]	1.14	1.00[h]	11.32	13.27
		325510W	1933890		1.14	1.00[h]	1.95	
	Aerosol Coatings	3255107 (10%)[f]	671641	4.616[g]	1.14	1.00[h]	0.39	0.39
	Allied Paint Products	325510B	1383897	2.987[g]	1.14	1.00[h]	1.26	1.26
	Industrial Coatings	3255104	6157635	4.310[g]	1.14	1.00[h]	3.87	7.42
		3255107 (90%)[f]	6044765	4.616[g]	1.14	1.00[h]	3.55	
Printing Inks	Printing Inks	3259101	265917	4.217	1.17	1.07	0.18	3.20
		3259104	1050099		1.17	1.07	0.71	
		3259107	271753		1.17	1.07	0.18	
		325910A	837641		1.17	1.07	0.56	
		325910E	1233022		1.17	1.07	0.83	
		325910H	608350		1.17	1.07	0.41	
		325910W	498817		1.17	1.07	0.34	
Pesticides & FIFRA Products	FIFRA Pesticides	3253204	905062	4.552	1.05	1.04	0.61	1.46
		3253207	1273582		1.05	1.04	0.86	
	Agricultural Pesticides	3251994	1475958	4.552	1.04	1.12	1.08	10.32
		3253201	12842492		1.05	1.04	8.63	
		325320W	911325		1.05	1.04	0.61	
Dry Cleaning	Dry Cleaning	[i]						0.03
Oil & Gas	Oil & Gas							1.32
Misc. Products	Misc. Products	[j]						0.18
Fuels & Lighter	Fuels & Lighter							2.80

[a]: All values ($) retrieved via the U.S. Census Bureau ASM's API tool.

[b]: All quantities retrieved from Table 6 of U.S. Department of Transportation (2015) and representative of 2012 values.

[c]: U.S. Bureau of Labor Statistics, Producer Price Index by Industry, retrieved from FRED, Federal Reserve Bank of St. Louis. All re-indexed to 2010.

[d]: Annual usage $[\text{kg/person/year}] = (\textbf{\textit{ASM Shipment Values}}) \div \left(\textbf{\textit{Commodity Price}} \times \left({}^{\textbf{2016 \textit{Index}}}/_{\textbf{2012 \textit{Index}}}\right)\right) \div (\textbf{\textit{Population}})$

[e]: NAICS code 3256207 includes all hair preparation products. Based on sales data by California Air Resources Board 2015 Consumer and Commercial Products Survey Data, we estimate ~75% of all hair preparation products are short-use (e.g. shampoos, conditioners) and ~25% are daily-use (e.g. hair spray, other leave-in products).

[f]: NAICS code 3255107 includes all special-purpose coating materials (e.g. automotive finishing, traffic markings, aerosolized painting products). Based on shipment data from U.S. Census Bureau, Paint and Allied Products - 2010, MA325F(10), Issued July 2011, we estimate ~10% of all special-purpose coating materials in this NAICS are aerosolized painting products, with the residual consisting of special-purpose coating materials used exclusively in industrial settings.

[g]: Commodity values retrieved from: U.S. Census Bureau, Paint and Allied Products - 2010, MA325F(10), Issued July 2011.

[h]: To be consistent with U.S. Census Bureau, Paint and Allied Products – 2010, producer price indices from 2010 used here.

[i]: sub-PUC usage estimated from solvent usage as reported by The Freedonia Group; Industry Study #3429; Solvents; July 2016.

[j]: sub-PUC usage estimated from reported sales data by California Air Resources Board 2015 Consumer and Commercial Products Survey Data (CARB, 2019).

Table S3: Derivation of complete (1st-order and organic) product composition profiles for Adhesives & Sealants. A similar composite tables was generated for all sub-PUCs,

Product Type	CARB Profile[a]	Sales [tpd]	Sales [%]	Water [%]	Inorganic [%]	Organic [%]	Evaporative Organics [%][b]	Evaporative Organics Profile Composite [%][c]
Other adhesives	3096	58.95	38.6%	3.97%	61.47%	34.56%	1.09%	16.81%
Sealant or Caulking Compound -- Nonchemically Curing	3005	30.34	19.9%	18.44%	51.43%	30.14%	2.14%	16.99%
Spackling Compound	3005	12.43	8.1%	19.58%	51.06%	29.36%	1.56%	5.07%
Carpet and Tile Adhesive	3003	11.69	7.7%	17.45%	52.61%	29.93%	1.29%	3.95%
Construction, Panel, or Floor Covering Adhesive	3001	8.81	5.8%	26.41%	44.08%	29.51%	5.52%	12.73%
Sealant or Caulking Compound -- Chemically Curing	3005	7.31	4.8%	1.05%	60.67%	38.28%	5.25%	10.04%
Other sealants and caulks	3005	6.85	4.5%	8.57%	55.92%	35.51%	5.07%	9.10%
Woodworking Glue	1510	6.00	3.9%	47.26%	33.39%	19.35%	1.17%	1.84%
General Purpose Adhesive	3002	4.40	2.9%	28.14%	41.66%	30.20%	7.52%	8.67%
Insulating and Sealing Spray Foam	3084	2.48	1.6%	0.10%	55.03%	44.87%	14.91%	9.69%
Wood Filler	1521	1.27	0.8%	12.63%	54.66%	32.70%	2.95%	0.98%
Arts and Crafts Adhesive	3105	0.66	0.4%	68.53%	18.91%	12.55%	2.26%	0.39%
Contact Adhesive - General Purpose	3002	0.50	0.3%	42.69%	34.97%	22.34%	3.30%	0.43%
Floor Seam Sealer	3001	0.24	0.2%	47.11%	30.29%	22.60%	6.11%	0.39%
Specialty Automotive Adhesive	1503	0.24	0.2%	0.13%	54.13%	45.75%	16.28%	1.02%
Pipe Thread Sealant/Pipe Joint Compound	3004	0.14	0.1%	0.00%	61.25%	38.74%	5.40%	0.20%
Contact Adhesive - Special Purpose	2513	0.08	0.1%	1.04%	22.16%	76.80%	64.73%	1.37%
Thread Locking Compound*	3002	0.06	0.0%	0.18%	58.07%	41.75%	10.14%	0.16%
Tile and Grout Sealer	3097	0.06	0.0%	70.33%	18.07%	11.61%	1.77%	0.03%
Household Glues and Paste	3002	0.05	0.0%	53.23%	24.11%	22.66%	9.54%	0.14%
**Adhesives & Sealants[d]**	**--**	**152.6**	**--**	**12.80%**	**53.22%**	**33.99%**	**5.02%**	**--**

[a]: Assigned organic profile for each product type. Retrieved from California Air Resources Board and available: https://ww2.arb.ca.gov/speciation-profiles-used-carb-modeling; last access: August 28, 2020

[b]: "Evaporative Organics" is a component of "Organic." This represents the potentially evaporative organic fraction and excludes "non-evaporative" (i.e. non-volatile) organics, which are not included in the California Air Resource Board's organic profiles.

[c]: Percent of "Evaporative Organic," weighted by sales abundance:

$$\boldsymbol{Evaporative\ Organic\ Profile\ Composite}\ [\%] = (\boldsymbol{Sales})_i \times (\boldsymbol{Evaporative\ Organic})_i \div \left(\sum_{i=1}^{n} (\boldsymbol{Sales})_i \times (\boldsymbol{Evaporative\ Organics})_i \right)$$

Where i is the Product Type index and n = 20 (i.e. all Product Types).

[d]: All water, inorganic, organic, and evaporative organics percentages for the complete sub-PUC are derived on a weighted basis from the reported sales abundance.

Table S4: Organic composition profile source/method summary for all sub-PUCs.

Product Use Categories (PUCs)	Sub-Product Use Categories (sub-PUCs)	Organic Composition Source/Methods
Cleaning Products	Detergents & Soaps	Product type composite derived from CARB's 2015 Consumer and Commercial Products Survey[b] and speciated using CARB organic profiles[c]
	General Cleaners	
Personal Care Products	Daily Use Products	
	Short Use Products	
Adhesives & Sealants	Adhesives & Sealants	
Paints & Coatings	Architectural Coatings	Product type composite derived from CARB's 2005 Architectural Coatings Survey[d] (Assumes 94% is water-based, 6% is solvent-based[e]) and speciated using CARB organic profiles[c].
	Aerosol Coatings	Product type composite derived from CARB's 2010 Aerosol Coatings Survey[f] and speciated using CARB organic profiles[c]
	Allied Paint Products	Product type composite derived from CARB's 2015 Consumer and Commercial Products Survey[b] and speciated using CARB organic profiles[c]
	Industrial Coatings	SPECIATEv5.0[g] Profile: 3149
Printing Inks	Printing Inks	SPECIATEv5.0[g] Profile: 2570
Pesticides & FIFRA Products	FIFRA Pesticides	Product type composite derived from CARB's 2015 Consumer and Commercial Products Survey[b] and speciated using CARB organic profiles[c]
	Agricultural Pesticides	
Dry Cleaning	Dry Cleaning	SPECIATEv5.0[g] Profile: 2422
Oil & Gas	Oil & Gas	[a]
Misc. Products	Misc. Products	Product type composite derived from CARB's 2015 Consumer and Commercial Products Survey[b] and speciated using CARB organic profiles[c]
Fuels & Lighter	Fuels & Lighter	

[a]: According to The Freedonia Group; Industry Study #3429; Solvents; July 2016: ~65% of all solvents used in O&G operations are alcohols, with the residual largely consisting of "hydrocarbons." We allocate all alcohols to methanol, as it is widely used in and emitted from O&G operations (Stringfellow, et al., 2017; Lyman et al., 2018; Mansfield et al., 2018). We treat the remaining 35% as naphtha, a blend of hydrocarbon solvents.

[b]: Ref: CARB, 2019

[c]: Ref: CARB, 2018

[d]: Ref: CARB, 2007

[e]: Ref: CARB, 2014

[f]: Ref: CARB, 2012

[g]: Ref: EPA, 2019b

Table S5: Assigned use timescales for all sub-PUCs.

Product Use Categories (PUCs)	Sub-Product Use Categories (sub-PUCs)	Use Timescale
Cleaning Products	Detergents & Soaps	Minutes
	General Cleaners	Days
Personal Care Products	Daily Use Products	Days
	Short Use Products	Minutes
Adhesives & Sealants	Adhesives & Sealants	Years
Paints & Coatings	Architectural Coatings	Years
	Aerosol Coatings	Years
	Allied Paint Products	Years
	Industrial Coatings	Years
Printing Inks	Printing Inks	Years
Pesticides & FIFRA Products	FIFRA Pesticides	Weeks
	Agricultural Pesticides	Weeks
Dry Cleaning	Dry Cleaning	Minutes
Oil & Gas	Oil & Gas	Years
Misc. Products	Misc. Products	Years
Fuels & Lighter	Fuels & Lighter	Years

Table S6: Methods and Data Sources for Allocating National Emissions to County-level.

Sub-Product Use Categories (sub-PUCs)	Allocation Proxy	Employment NAICS[a]	NAICS Description
Detergents & Soaps	Population	--	--
General Cleaners	Population	--	--
Daily Use Products	Population	--	--
Short Use Products	Population	--	--
Adhesives & Sealants	Population	--	--
Architectural Coatings	Population	--	--
Aerosol Coatings	Population	--	--
Allied Paint Products	Employment	236///	Construction of Buildings
Industrial Coatings	Employment	811121	Automotive Body, Paint, and Interior Repair and Maintenance
		4411//	Automobile Dealers
		4412//	Other Motor Vehicle Dealers
		336411	Aircraft Manufacturing
		3365//	Railroad Rolling Stock Manufacturing
		3366//	Ship and Boat Building
		488390	Other Support Activities for Water Transportation
		339///	Miscellaneous Manufacturing
		3369//	Other Transportation Equipment Manufacturing
		811///	Repair and Maintenance
		3133//	Textile and Fabric Finishing and Fabric Coating Mills
		332812	Metal Coating, Engraving
		2373//	Highway, Street, and Bridge Construction
		321///	Wood Product Manufacturing
		337110	Wood Kitchen Cabinet and Countertop Manufacturing
		337121	Upholstered Household Furniture Manufacturing
		337122	Non-upholstered Wood Household Furniture Manufacturing
		337211	Wood Office Furniture Manufacturing
		337212	Custom Architectural Woodwork and Millwork Manufacturing
		337124	Metal Household Furniture Manufacturing
		337214	Office Furniture (except Wood) Manufacturing
		337215	Showcase, Partition, Shelving, and Locker Manufacturing
		322///	Paper Manufacturing
		325992	Photographic Film, Paper, Plate, and Chemical Manufacturing
		33243/	Metal Can, Box, and Other Metal Container Manufacturing
		333///	Machinery Manufacturing
		3352//	Household Appliance Manufacturing
		331318	Other Aluminum Rolling, Drawing, and Extruding
		3314//	Nonferrous Metal (except Aluminum) Production and Processing
		33592/	Communication and Energy Wire and Cable Manufacturing
		335311	Power, Distribution, and Specialty Transformer Manufacturing
		3361//	Motor Vehicle Manufacturing
		3362//	Motor Vehicle Body and Trailer Manufacturing
		3363//	Motor Vehicle Parts Manufacturing
Printing Inks	Employment	32311/	Printing
		3222//	Converted Paper Product Manufacturing
FIFRA Pesticides	Population	--	--
Agricultural Pesticides	Pesticide Use	[b]	
Dry Cleaning	Employment	812320	Dry Cleaning and Laundry Services
Oil & Gas	O&G Well Count	[c]	
Misc. Products	Population	--	--
Fuels & Lighter	Population	--	--

[a]: All employment mapping, expect Allied Paint Products, follows the NAICS mapping from the 2017 NEI (U.S. EPA, 2017). For Allied Paint Products, mapping is allocated based on construction employment.

[b]: Allocation of Agriculture Pesticides emissions follows the mapping from the 2017 NEI (U.S. Geological Survey, Pesticide National Synthesis Project, https://water.usgs.gov/nawqa/pnsp/usage/maps/county-level/; last access: August 31, 2020).

[c]: U.S. Energy Information Administration, The Distribution of U.S. Oil and Natural Gas Wells by Production Rate, Washington, DC, 2019.

Table S7: Observed emission ratios (de Gouw et al., 2017; de Gouw et al., 2018) and inventory emission ratios for Los Angeles County. VCPy: All emissions retrieved from the 2017 NEI, except VCPs, which are replaced using the emissions derived in this study (representative of 2016). 2017 NEI: All emissions retrieved from the 2017 NEI. Emissions consist of all on-road, non-road, non-point, and point sources, as well as biogenic ethanol, methanol, and acetone. Total CO emissions (~320 Gg) include all on-road, non-road, non-point, and point sources.

Compound	Observed [g /g CO]	VCPy [g /g CO]	2017 NEI [g /g CO]
Ethanol	0.0752	0.0567	0.0301
Acetone	0.0241	0.0265	0.0116
i-Propanol	0.0212	0.0134	0.0070
Toluene	0.0112	0.0227	0.0169
Propane	0.0211	0.0117	0.0074
i-Butane	0.0066	0.0079	0.0060
(m + p)-Xylenes	0.0078	0.0090	0.0041
n-Butane	0.0103	0.0141	0.0116
Methyl Ethyl Ketone	0.0023	0.0044	0.0017
Methanol	0.0243	0.0187	0.0172
Undecane	0.0018	0.0030	0.0012
Octanes	0.0072	0.0031	0.0014
Heptane	0.0030	0.0026	0.0021
Nonane	0.0015	0.0018	0.0006
Hexane	0.0043	0.0045	0.0039
Methylcyclohexane	0.0015	0.0015	0.0007
Trimethylbenzenes	0.0063	0.0032	0.0022
Ethyltoluenes	0.0024	0.0030	0.0020
Decane	0.0015	0.0012	0.0004
Ethylbenzene	0.0023	0.0025	0.0019
n-Pentane	0.0088	0.0046	0.0043
Dimethylcyclohexanes	0.0008	0.0004	0.0001
Styrene	0.0013	0.0013	0.0013
Propylbenzenes	0.0006	0.0007	0.0004
o-Xylene	0.0029	0.0019	0.0018
Methylacetate	0.0006	0.0003	0.0001
2-Methylhexane	0.0021	0.0012	0.0011
n-Propanol	0.0007	0.0002	0.0001
3-Methylpentane	0.0043	0.0022	0.0022
Cyclohexane	0.0016	0.0018	0.0017

Table S8: SCC – SPECIATEv5.0 (EPA, 2019b) profile mapping for all non-point sources in the 2017 NEI. A similar mapping scheme was used to additionally speciate 53 on-road SCCs, 57 non-road SCCs, and > 4,500 point SCCs.

SCC	SPECIATE Profile	SCC	SPECIATE Profile	SCC	SPECIATE Profile	SCC	SPECIATE Profile
2401001000	95513	2104006000	0195	2620030000	3002	2810005001	5560
2401005000	2402	2104007000	0195	2102004001	0002	2810035000	5560
2401008000	3135	2302002100	4553	2102004002	0002	2104002000	1185
2401090000	3149	2302002200	4553	2102007000	0003	2601020000	0122
2401100000	3138	2302003000	4652	2304000000	1089	2103002000	1178
2401200000	3138	2302003100	4651	2308000000	1008	2810005000	5560
2420000000	2422	2302003200	4651	2309000000	2466	2830000000	0000
2425000000	1191	2501011011	8870	2312000000	0000	2505020180	2488
2460100000	95509	2501011012	8870	2399000000	0000	2302070000	1188
2460200000	95508	2501011013	8870	2510000000	0000	2862000000	0000
2460400000	95510	2501011014	8870	2620000000	3002	2301000000	2462
2460500000	95512	2501011015	8870	2650000000	3002	2301030000	2462
2460600000	95507	2501012011	8870	2660000000	8870	2635000000	8870
2460800000	95511	2501012012	8870	2810030000	0000	2840000000	2402
2460900000	95512	2501012013	8870	2810040000	5565	2851001000	0000
2461021000	1007	2501012014	8870	2810050000	0000	2501070053	DIESEVP
2461022000	1007	2501012015	8870	2830001000	0000	2810003000	4659
2461850000	CARB3103	2501060053	8870	2102001000	1185	2601000000	122
2415000000	8745	2501060201	8870	2102011000	0004	2601010000	122
2401015000	2405	2501080050	8869	2501060052	8870	2630010000	3003
2401020000	2405	2501080100	8869	2103004000	0002	2302070010	1188
2401025000	2406	2505030120	8870	2301010000	2462	2680002000	0000
2401055000	3149	2610000100	0121	2302000000	4553	2306010100	0026
2401075000	2414	2610000400	0121	2302050000	1188	2620010000	3002
2401080000	2415	2610000500	0121	2302070005	1188	2102010000	0004
2401070000	3131	2610030000	0121	2302080000	1188	2310010100	0003
2401030000	2552	2630020000	3003	2306010000	0026	2310010200	2487
2401040000	2408	2680003000	8933	2505010000	2489	2310011001	1011
2401060000	2411	2810025000	4553	2505020000	0305	2310011201	2487
2401065000	3138	2810060100	0000	2610000300	0121	2310011501	1011
2401085000	2416	2810060200	0000	2640000000	0000	2310011502	1011
2440000000	95512	2104004000	0002	2680001000	0000	2310011503	1011
2401005700	2402	2104011000	0002	2102005000	0001	2310011505	1011
2401010000	3137	2501050120	8869	2302002000	4553	2310021010	2487
2420000999	2422	2501055120	8869	2305000000	0000	2310021030	2487
2461023000	1007	2505040120	8869	2307000000	2405	2310021100	0003
2401050000	3127	2102002000	1185	2325030000	0000	2310021300	8949
2420000055	0085	2102006000	0003	2501995120	8762	2310021302	1001
2401045000	2409	2102008000	4642	2302070001	1188	2310021351	1001
2425010000	2543	2103004001	0002	2301020000	1092	2310021400	0003
2425020000	2544	2103004002	0002	2801520000	3161	2310021501	8949
2425030000	2545	2103007000	0003	2103005000	0001	2310021502	8949
2425040000	1086	2103008000	4642	2103001000	1178	2310021503	8949
2461020000	1007	2103011000	0002	2520010000	0000	2310021505	8949
2461800001	CARB3103	2302010000	4553	2501080201	8762	2310021506	8949
2461800002	CARB3103	2501060051	8870	2505020030	0305	2310021603	8949
2440020000	95507	2102004000	0002	2505020060	0305	2310023300	8950
2460000000	95512	2103006000	0003	2505020090	2488	2310023302	1001
2401035000	3137	2311030000	0000	2505020120	8869	2310023351	1001

2461100000	0000
2310023511	8950
2310023512	8950
2310023513	8950
2310023515	8950
2310023516	8950
2310000220	0008
2310111100	1011
2310111700	1011
2310000660	0008
2310023600	8950
2310121700	8949
2310001000	8949
2310010300	8949
2310021251	1001
2310000553	1207
2310011600	1001
2310021202	1001
2310111401	1011
2310121401	8949
2310121100	8949
2310021303	1001
2310022420	8949
2310002401	8949

2325000000	0000
2310000552	1207
2310023100	0003
2310023202	1001
2310023251	1001
2310023310	8950
2310023603	8950
2310011500	95399
2310020000	8949
2310022000	8949
2310010700	DJVNT_R
2310011450	DJVNT_R
2310021310	DJVNT_R
2310021509	DJVNT_R
2310021700	1001
2310030220	FLR99
2310030300	1207
2310030400	2487
2310111701	FLR99
2310321603	DJVNT_R
2310400220	0008
2310022010	95109a
2310022090	0003
2310022506	1010

2505020150	100
2310421100	0003
2310011020	2487
2310021109	1001
2310021209	1001
2310021309	1001
2310021600	SSJCO_R
2310021602	SSJCO_R
2310000551	1207
2310023010	2487
2310023030	2487
2310021102	1001
2310023606	SSJCB_R
2310023509	8950
2310023102	1001
2310021500	FLR99
2310300220	0008
2310321010	2487
2310321100	0003
2310321400	0003
2310421010	2487
2310021802	95417
2310021801	95417
2310021803	FLR99

2310023400	0003
2310421603	8949
2310421400	0003
2310020600	1001
2310011504	1011
2310011506	1011
2310021103	1001
2310021402	1001
2310021403	1001
2310021450	8949
2310021504	8949
2310021101	1001
2310021203	1001
2310021301	1001
2310002000	8949
2310002421	8949
2310012000	1011
2310012020	95087a
2310012526	1011
2310022105	0008
2310112401	1011
2310011100	0003
2310000230	0008

95

Table S9: National-level emission rates [kg person^{-1} year^{-1}] for the top-200 compounds emitted from VCPs, as predicted by VCPy.

Compound	Emissions	Compound	Emissions	Compound	Emissions	Compound	Emissions
Ethanol	1.6519	C6 Cycloalkanes	0.0238	C10 Trialkylbenzenes	0.0052	cis-1,cis-3,5-trimethylcyclohexane	0.0022
Acetone	0.8506	d-Limonene	0.0235	Aliphatics	0.0052	trans,cis-1,2,4-trimethylcyclohexane	0.0022
Isopropyl Alcohol	0.4274	C15 Cycloalkanes	0.0223	Isobutyl Acetate	0.0051	1,1,3-trimethylcyclohexane	0.0022
Toluene	0.3704	Other, Misc. VOC Compounds Aggregated In Profile	0.0219	C11 Tetrasubstituted Benzenes	0.0051	1,1,3-trimethylcyclopentane	0.0022
n-Tetradecane	0.3632	Branched C10 Alkanes	0.0208	2,6-dimethylnonane	0.0050	4-methyldecane	0.0022
Fragrances	0.3444	C10 Cycloalkanes	0.0208	Methyl Acetate	0.0050	1,2,3-Trimethylbenzene	0.0021
Propane	0.3365	Witch Hazel	0.0197	3-methylheptane	0.0049	5-methyldecane	0.0021
Volatile Methyl Siloxanes	0.3024	2-Amino-2-Methyl-1-Propanol	0.0188	2-methylhexane	0.0049	trans,trans-1,3,5-trimethylcyclohexane	0.0021
Isobutane	0.2814	n-Tridecane	0.0186	1-Tetradecene	0.0049	Butylcyclohexane	0.0021
Propylene Glycol	0.2488	n-Heptane	0.0167	C15 Branched Alkanes	0.0048	4-methylheptane	0.0021
2,2,4-Trimethyl-1,3-Pentanediol Isobutyrate (Texanol)	0.2195	Hexane	0.0162	Other, Lumped VOCs, Individually < 2% Of Category	0.0047	2,3-Dimethylbutane	0.0021
Ethylene Glycol	0.2144	Propylene Glycol Monomethyl Ether Acetate	0.0159	1,2,4-trimethylcyclopentane	0.0046	C11 Tetralin or Indane	0.0020
Xylenes	0.1941	Benzene	0.0155	1,2-dimethylcyclopentane	0.0046	Trichloroethylene	0.0020
n-Butane	0.1916	C7 Cycloalkanes	0.0151	Turpentine	0.0046	Diisopropyl Adipate	0.0020
Methanol	0.1772	C14 Cycloalkanes	0.0150	Diethylene Glycol	0.0045	1,3-diethylbenzene (meta)	0.0019
Ethylene Glycol Monobutyl Ether	0.1588	n-Pentane	0.0149	C12 Naphthalenes	0.0045	2,2-Dimethylbutane	0.0018
Branched C12 Alkanes	0.1568	Branched C7 Alkanes	0.0146	Propylene Glycol Monomethyl Ether (1-Methoxy-2-propanol)	0.0045	Dihydroxyacetone	0.0018
n-Undecane	0.1461	Ethyl Cyanoacrylate	0.0139	n-Propyl Alcohol	0.0044	Dipropylene Glycol	0.0017
Methylene Chloride (Dichloromethane)	0.1291	C13 Cycloalkanes	0.0129	trans,trans-1,2,4-trimethylcyclohexane	0.0043	Dimethyl Succinate	0.0016
Methyl Ethyl Ketone (2-Butanone)	0.1287	Isopropyl acetate	0.0126	Diethylene Glycol Monoethyl Ether	0.0043	Ethylene Glycol Monopropyl Ether	0.0015
Dimethyl Ether	0.1186	Propylene Glycol N-Propyl Ether	0.0124	C5 Branched Alkanes	0.0042	Triethanolamine	0.0015
n-Dodecane	0.1100	1,1,1,2-Tetrafluoroethane (HFC-134a)	0.0124	Hexadecane	0.0040	p-Xylene	0.0015
1,1-Difluoroethane (HFC-152a)	0.1082	Hydrocarbon Propellant (LPG)	0.0122	m-Xylene	0.0040	C11 Dialkyl Benzenes	0.0014
Hydrocarbon Propellant (LPG, Sweetened)	0.0914	Cyclopentane	0.0118	Branched C17 Alkanes	0.0040	C14 Branched Alkanes	0.0014
C11 Cycloalkanes	0.0898	N,N-Diethyl-M-Toluamide	0.0115	Methyl Amyl Ketone	0.0040	Methyl Ethyl Ketoxime	0.0014
n-Octane	0.0803	Styrene	0.0112	1,2,4-Trimethylbenzene	0.0038	2,2,4,6,6-Pentamethylheptane	0.0014
C12 Cycloalkanes	0.0758	C16 Cycloalkanes	0.0109	Misc. Oxygenated Compounds	0.0037	(2-methylpropyl)benzene (or isobutylbenzene)	0.0014
C13 Branched Alkanes	0.0747	1,3,5-trimethylbenzene	0.0107	C10 Dialkyl Benzenes	0.0035	Toluene	0.0013
Branched C9 Alkanes	0.0700	N-Methylpyrrolidinone	0.0107	Dipropylene Glycol Monopropyl Ether	0.0034	Methyl Propyl Ketone (2-Pentanone)	0.0013

C8 Cycloalkanes	0.0617	Aggregated Vocs < 1.0%	0.0104	Phenoxyethanol	0.0033	Methyltriacetoxysilane	0.0013
Pine Oil	0.0605	C11 Trialkyl Benzenes	0.0101	Diethanolamine	0.0033	Ethyltriacetoxysilane	0.0012
n-Nonane	0.0551	Benzyl Alcohol	0.0099	m-Xylene	0.0032	Hexylene Glycol (2-Methyl-2,4-Pentanediol)	0.0012
Branched C11 alkanes	0.0538	C12 Trisubstituted Benzenes	0.0092	Diisobutyl Ketone	0.0031	Isobutyl Alcohol	0.0012
Ethyl Acetate	0.0481	cis-1,3-dimethylcyclohexane	0.0090	3-Methylpentane	0.0030	Ethylbenzene	0.0012
Perchloroethylene (Tetrachloroethene)	0.0481	White Mineral Oil	0.0089	Methyl Methacrylate	0.0030	1,2-diethylbenzene (ortho)	0.0012
C9 Cycloalkanes	0.0474	Diethylene Glycol Monomethyl Ether	0.0086	1-Ethyl-2-Propyl Cyclohexane	0.0029	Diacetone Alcohol	0.0012
n-Decane	0.0445	o-Xylene	0.0085	Dimethyl Adipate	0.0028	Tetramethylbenzenes	0.0012
n-Heptane	0.0422	n-Pentadecane	0.0083	Ethylcyclohexane	0.0028	1,3,5-Trimethylbenzene	0.0011
Branched C8 Alkanes	0.0407	2-Methylheptane	0.0076	trans-1,4-dimethylcyclohexane	0.0027	Misc. Hydrocarbon Propellants	0.0011
Ethanolamine	0.0381	C16 Branched Alkanes	0.0072	Cumene	0.0027	1,4-diethylbenzene (para)	0.0010
Methylcyclohexane	0.0369	o-Ethyltoluene	0.0072	trans-1,3-dimethylcyclohexane	0.0027	Diisopropylene glycol	0.0010
Dipropylene Glycol Monomethyl Ether	0.0307	2-Methylpentane	0.0071	4-methylnonane	0.0026	C13 Naphthalenes	0.0010
Branched C6 Alkanes	0.0306	Voc Ingredients < 0.1%	0.0068	Cyclohexane	0.0026	Ethylene Glycol Monohexyl Ether	0.0010
n-Hexane	0.0302	Glycerol	0.0067	2-methyldecane	0.0024	Tetrahydrofuran	0.0010
Diethylene Glycol Monobutyl Ether	0.0297	n-Propylbenzene	0.0065	2-Ethylhexyl Benzoate	0.0024	Cyclohexanol	0.0010
Ethyl Benzene	0.0286	Propylene Glycol Butyl Ether (1-Butoxy-2-Propanol)	0.0063	trans 1-methyl-3-propyl cyclohexane	0.0024	Methylindans	0.0010
m-Ethyltoluene	0.0281	1,2,3-trimethylbenzene	0.0059	3-methyldecane	0.0023	Diethyl Phthalate	0.0010
1,2,4-Trimethylbenzene	0.0270	Ethyl-3-Ethoxypropionate	0.0056	2,6-dimethylheptane	0.0023	Isopentane	0.0010
N-Butyl Acetate	0.0253	Glycol Ether Dpnb (1-(2-Butoxy-1-Methylethoxy)-2-Propanol)	0.0056	Misc. Esters	0.0023	Triethylene Glycol	0.0009
Methyl Isobutyl Ketone (Hexone)	0.0248	n-Butyl Alcohol	0.0055	Butyl Acrylate	0.0022	Pentanedioic Acid, Dimethyl Ester	0.0009

Table S10: Tabulation of Fig. 4 from main text.

Product Use Categories (PUCs)	2.5th	25th	Mean	75th	97.5th
Cleaning Products	1.25	1.72	2.02	2.3	2.94
Personal Care Products	1.32	1.76	2.01	2.25	2.81
Adhesives & Sealants	0.51	0.66	0.76	0.85	1.05
Paints & Coatings	2.53	2.92	3.12	3.34	3.77
Printing Inks	0.54	0.7	0.8	0.89	1.1
Pesticides & FIFRA Products	0.40	0.52	0.58	0.65	0.78
Other	0.02	0.06	0.16	0.24	0.46
Total	8.07	8.98	9.46	9.96	10.94

Table S11: Mapping of all sub-PUCs to equivalent Source Classification Codes (SCCs).

PUC	SCC	SCC Description	VCPy sub-PUC
Cleaning Products	2460200000	All Household Products	Detergents & Soaps<br>General Cleaners
	2415000000	Degreasing	
	2460400000	All Automotive Aftermarket Products	
Personal Care Products	2460100000	All Personal Care Products	Daily Use Products
			Short Use Products
Adhesives & Sealants	2460600000	All Adhesives and Sealants	Adhesives and Sealants
Paints & Coatings	2401001000	Architectural Coatings	Architectural Coatings
	2460500000	All Coatings and Related Products	Aerosol Coatings
	2401005000	Auto Refinishing	Industrial Coatings
	2401008000	Traffic Markings	
	2401015000	Factory Finished Wood	
	2401020000	Wood Furniture	
	2401025000	Metal Furniture	
	2401030000	Paper	
	2401040000	Metal Cans	
	2401055000	Machinery and Equipment	
	2401060000	Large Appliances	
	2401065000	Electronic and Other Electrical	
	2401070000	Motor Vehicles	
	2401075000	Aircraft	
	2401085000	Railroad	
	2401080000	Marine	
	2401090000	Misc. Manufacturing	
	2401100000	Industrial Maintenance Coatings	
	2401200000	Other Special Purpose Coatings	
	2402000000	Paint Strippers	Allied Paint Products
Printing Inks	2425000000	Graphic Arts, employment	Printing Inks
Pesticides & FIFRA Products	2460800000	All FIFRA Related Products	FIFRA Pesticides
	2461850000	Pesticide Application: Agricultural	Agricultural Pesticides
Dry Cleaning	2420000000	Dry Cleaning	Dry Cleaning
Oil & Gas	n/a	n/a	Oil & Gas
Misc. Products	2460900000	Miscellaneous Products: NEC	Misc. Products
Fuels and Lighter	n/a	n/a	Fuels and Lighter

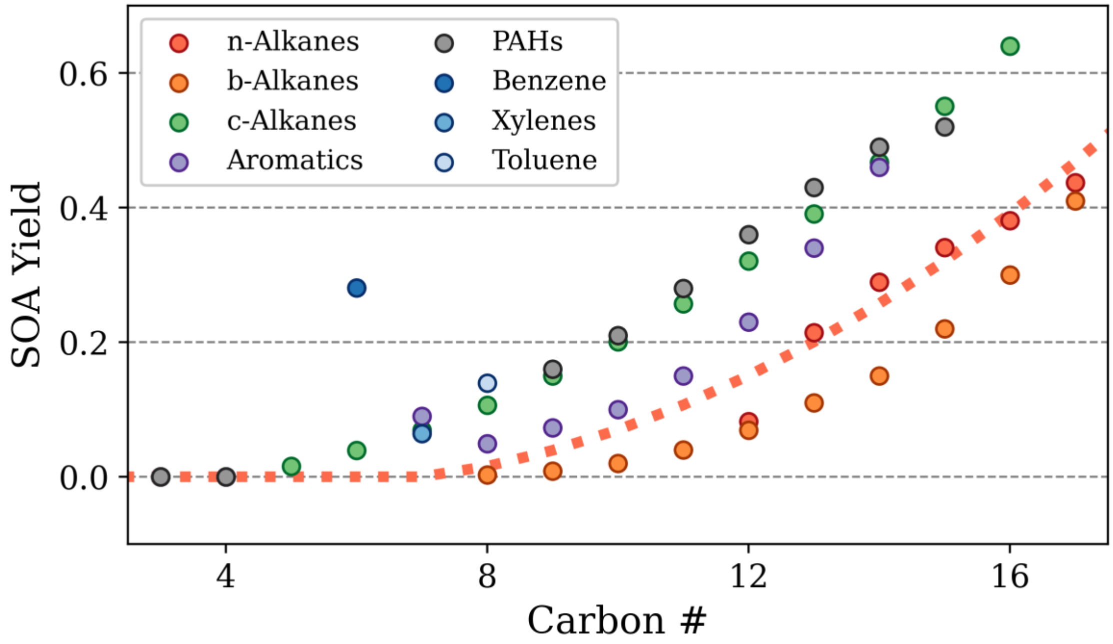

105 **Figure S1: Summary of SOA yields by compound class. Several references are used to construct these values and are described in the main text.**

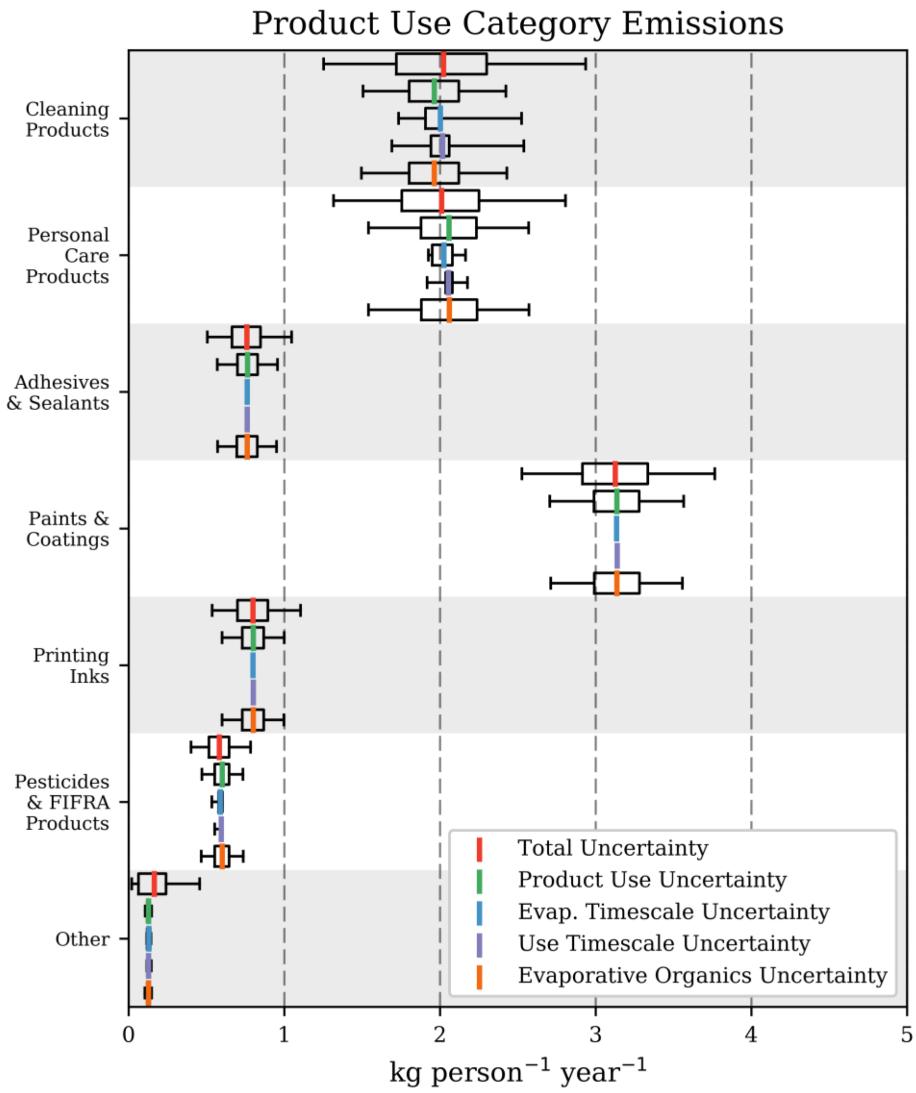

Figure S2: Monte Carlo sensitivity results: mean, interquartile range, and 95% confidence intervals for emission rates for six major PUCs and the sum of all others. Red: Estimate considering uncertainty in product usage, evaporation timescale, use timescale, and controls. Green: MC simulations that only perturb product usage uncertainties. Blue: MC simulations that only perturb evaporation timescale uncertainties. Purple: MC simulations that only perturb use timescale uncertainties. Orange: MC simulations that only perturb evaporative organic uncertainties.

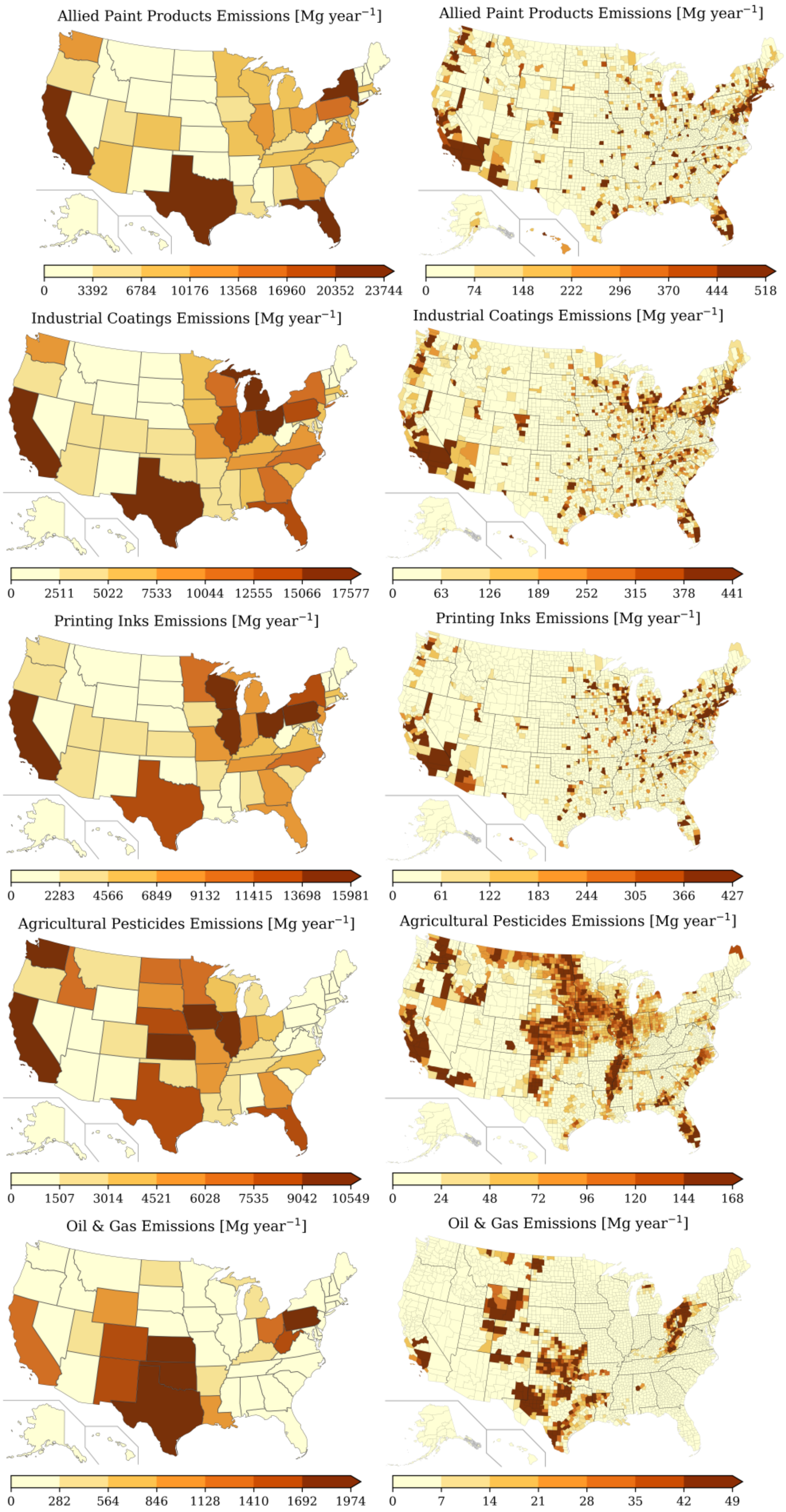

Figure S3: State- and County-level emissions for select sub-PUCs.

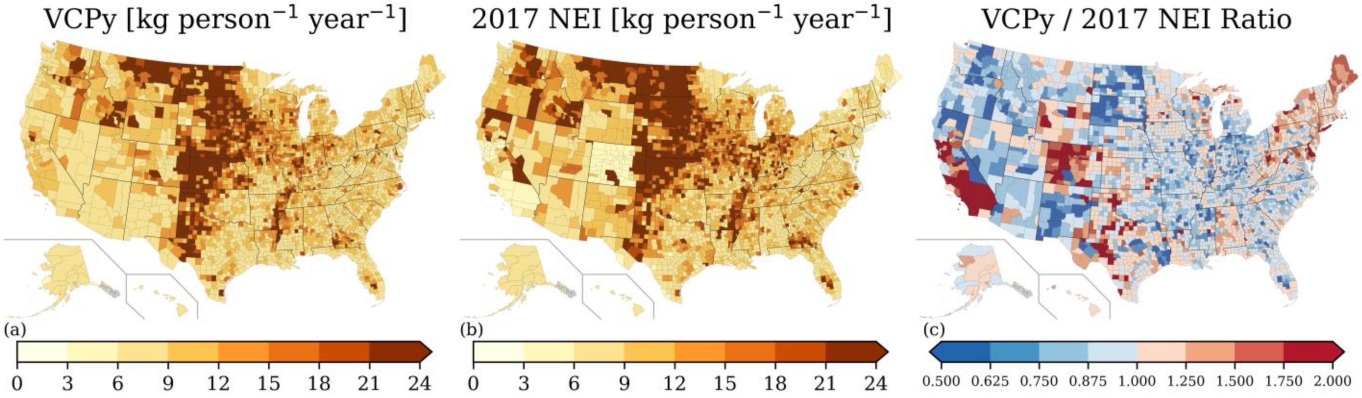

Figure S4: (a) County-level per-capita VCP emissions from the VCPy inventory (same as right panel of Fig. 5 in main text), (b) County-level per-capita VCP emissions from the 2017 NEI, and (c) County-level ratio of VCPy / 2017 NEI VCP emissions.

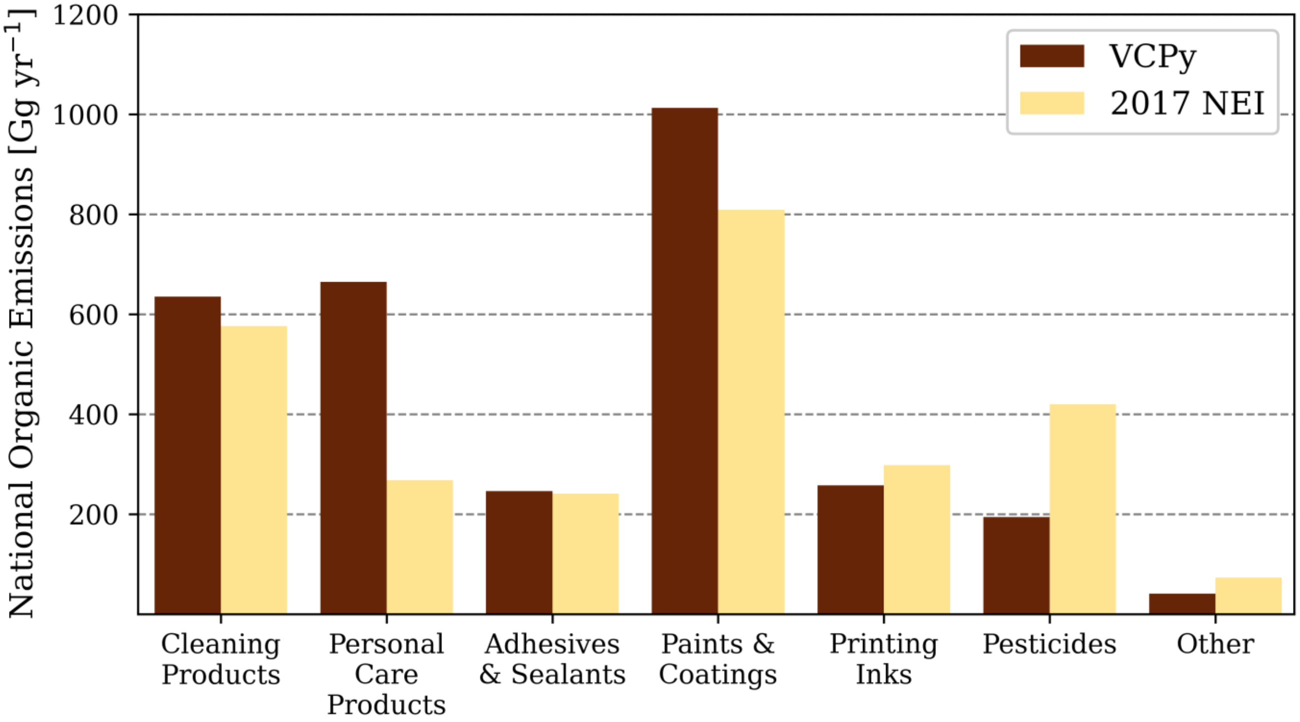

Figure S5: Product Use Category comparison of national-level emissions from the VCPy and 2017 NEI inventories for VCPs. For "Other," asphalt emissions in the 2017 NEI are excluded as those emissions are not quantified in the VCPy inventory.

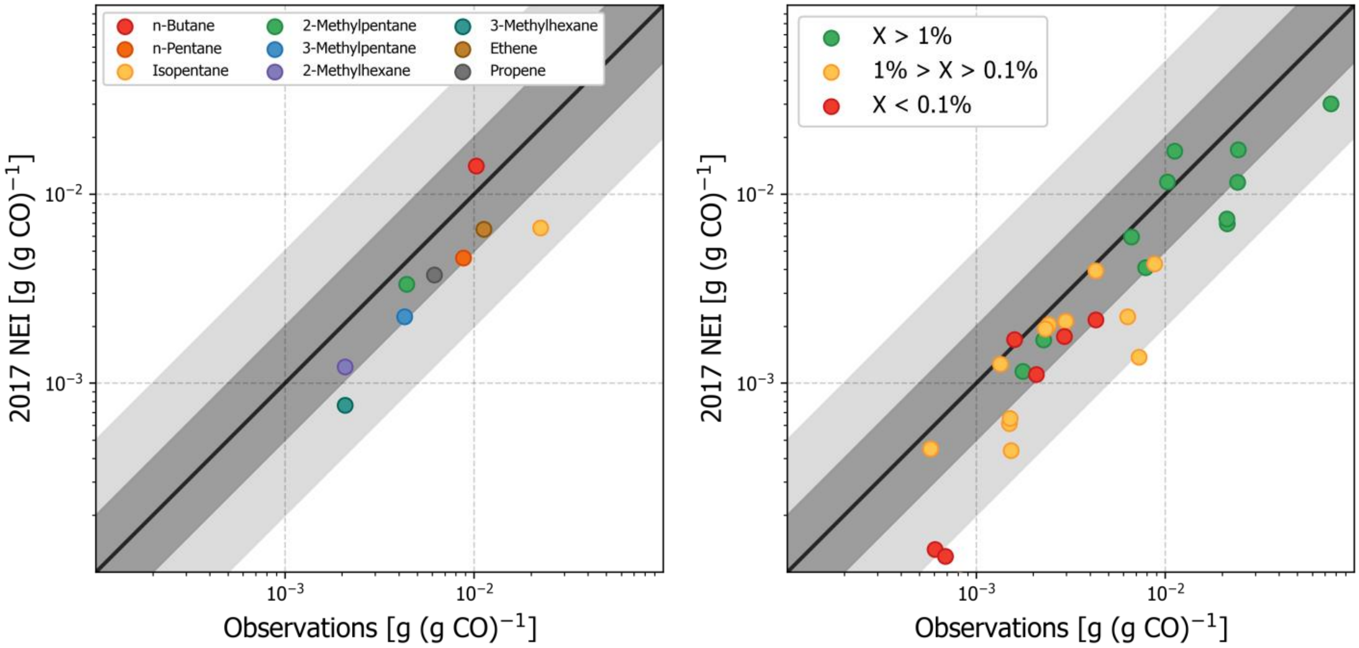

Figure S6: (Left) Evaluation of organic emission ratios of species that feature high emission factors from mobile sources in Los Angeles County using observed emission ratios from summer 2010. (Right) Evaluation of 2017 NEI organic emission ratios in Los Angeles County using observed emission ratios from summer 2010. The scatter point colors represent the relative abundance of each compound in the complete VCP sector. For example, all green points represent compounds that are > 1% of the total VCP emissions in Los Angeles County. Black line – 1:1; Dark grey shading – 2:1; Light grey shading – 5:1.

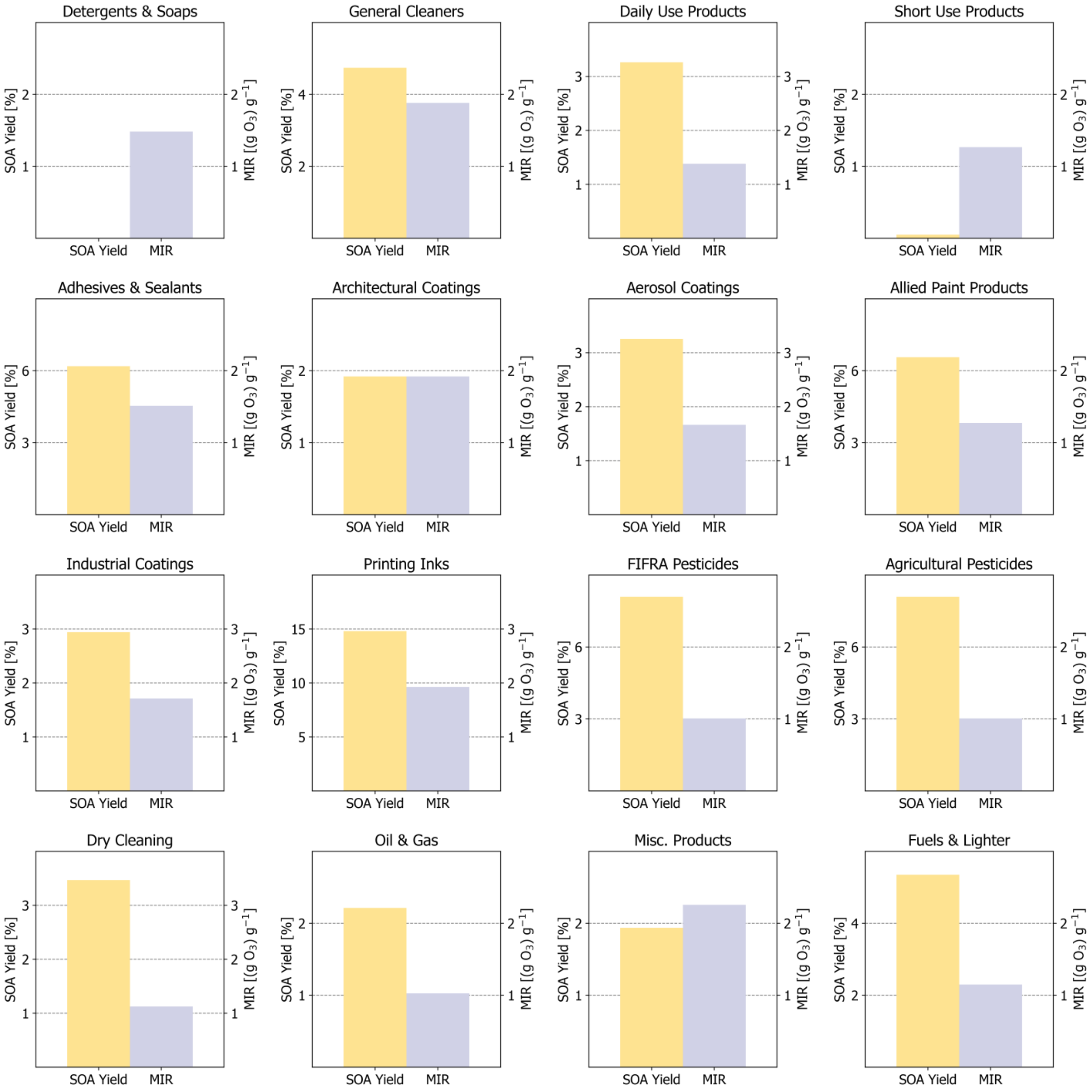

Figure S7: Effective SOA yield and MIR for all sub-PUCs.

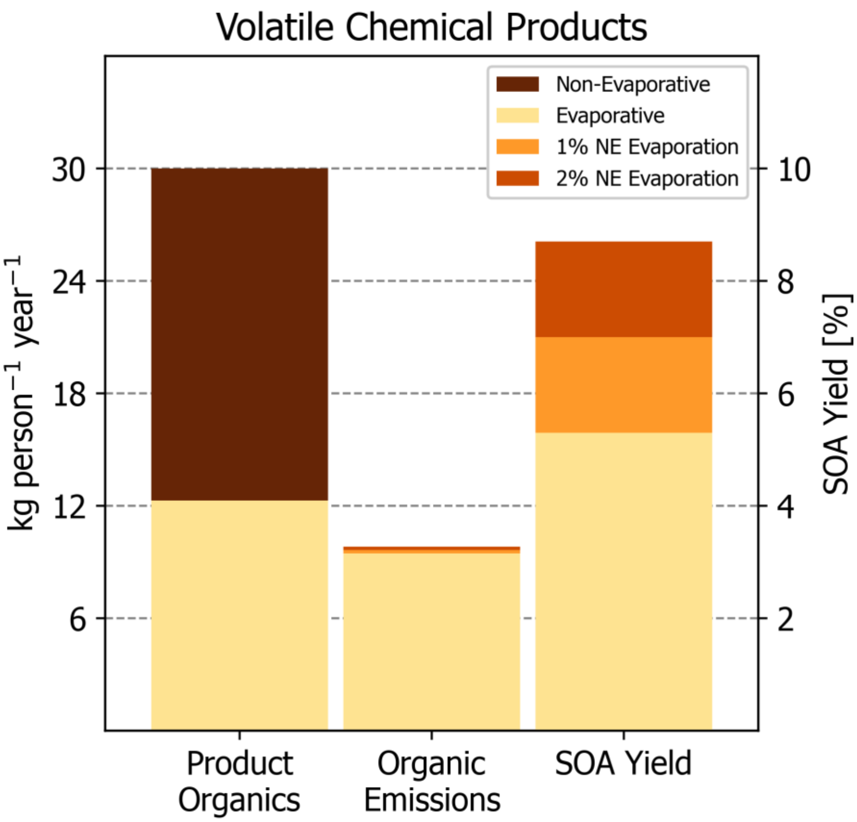

Figure S8: Total product organics, organic emissions, and sector-wide effective SOA yields resulting from adjusted non-evaporative assumptions. The two sensitivity tests are assuming 1% and 2% of all non-evaporative organic mass in VCPs evaporates and forms SOA with 100% efficiency.

145
